# Role of Tie‐2 Axis in Sepsis: A Potential Therapeutic Target

**DOI:** 10.1111/jcmm.70912

**Published:** 2025-10-22

**Authors:** Jun Feng, Junhui Liu, Xiaoyan Wu, Junshuai Wang

**Affiliations:** ^1^ Department of Emergency Medicine, Tongji Hospital, Tongji Medical College Huazhong University of Science and Technology Wuhan China; ^2^ Department of Critical Care Medicine, Tongji Hospital, Tongji Medical College Huazhong University of Science and Technology Wuhan China; ^3^ Tuberculosis I area Wuhan Pulmonary Hospital Wuhan China; ^4^ Department of Cancer Center, Union Hospital, Tongji Medical College Huazhong University of Science and Technology Wuhan China

**Keywords:** Ang–Tie‐2 axis, endothelial dysfunction, sepsis, therapeutic target

## Abstract

The angiopoietins (Angs)–Tie‐2 axis initiates signalling pathways that modulate vascular stability and angiogenesis and plays an important role in a variety of physiological and pathological processes, including inflammation, wound healing and cancer, by regulating endothelial cell proliferation, survival, migration, invasion and/or differentiation. Disruption of Ang‐1/2 and the Tie‐2 receptor can lead to endothelial cell activation or dysfunction, which can contribute to the pathogenesis of sepsis. Although the mechanisms by which the Ang–Tie‐2 axis participates in sepsis pathogenesis have not been fully elucidated due to the dynamic and complicated nature of sepsis, Ang–Tie‐2 axis malfunction causes endothelial cell activation and contributes to sepsis pathogenesis. During the initiation and development of sepsis, endothelial cells secrete Ang‐2, which inhibits Tie‐2 activation and subsequent signalling, leading to endothelial damage, increased vascular permeability, exacerbated inflammation and multiorgan injury. In this review, we summarise the latest advances in basic and clinical research on relevant papers from the PubMed database. We aim to offer a comprehensive overview of the current state of the art of the Ang–Tie‐2 axis in the context of sepsis and to explore the potential therapeutic targets for treating sepsis. A better understanding of the regulatory mechanisms related to the Ang–Tie‐2 axis could help identify potential therapeutic targets for treating sepsis.

## 1 Introduction

Sepsis can be caused by a variety of pathogen infections, and the host dysregulated immune response is a common pathogenesis that contributes to high morbidity and mortality [1, 2]. Sepsis has also been proposed as a syndrome of severe endothelial dysfunction, which emphasises the importance of endothelial cells in sepsis pathogenesis [3, 4]. Endothelial cells are vascular, unconventional immune cells involved in many important physiological functions. Based on pathophysiological responses characterised by hyperpermeability and the expression of cytokines, adhesion molecules and other key mediators, endothelial cells play a critical role in sepsis [5, 6]. In the course of sepsis, endothelial cells become activated, damaged or dysfunctional as a consequence of both the underlying infection and the overwhelming host response to infection, and severe endothelial dysfunction causes extensive damage to the lungs, kidneys, heart, brain and other organs, resulting in high mortality [7, 8, 9]. As a result, the central role of endothelial cells in the development of sepsis has attracted significant attention.

Accumulating evidence suggests that the disruption of angiopoietins (Angs) and the Tie‐2 receptor can lead to endothelial cell activation and potentially contribute to the pathogenesis of sepsis. Decreased Ang‐1 and increased Ang‐2 levels are hallmarks of sepsis [10, 11, 12]. Elevated Ang‐2 is associated with inflammation, increased cellular permeability and elevated intracellular gap formation. Nevertheless, administration of Ang‐1 can improve the inhibition of Ang‐1 and reduce the elevation of Ang‐2 induced by sepsis, thereby improving prognosis and reducing mortality [13, 14]. However, the specific mechanisms by which the Ang–Tie‐2 axis participates in endothelial dysfunction during sepsis have not been fully elucidated. In this review, we provide an overview of the Ang–Tie‐2 axis during sepsis and discuss its clinical significance in the pathophysiology of this complex biological syndrome. We also have attempted to establish the potential therapeutic targets for the management of sepsis. While further research is needed to fully understand the implications of the Ang–Tie‐2 axis in sepsis‐associated pathophysiology, it represents a promising new avenue of research.

## 2 Overview of the Ang–Tie‐2 Axis

Tie‐2 [where ‘Tie’ is an acronym for tyrosine kinase with Ig and EGF (epidermal growth factor) homology domains] is a receptor tyrosine kinase expressed predominantly on the surface of endothelial cells. Tie‐2 is a key regulator of vascular stability and angiogenesis and is involved in both inflammatory and coagulation responses in the blood [15, 16, 17]. The Angs family of growth factors includes four members, all of which bind to the endothelial receptor tyrosine kinase Tie‐2. Angs was first isolated and identified solely for its ability to induce the formation of new blood vessels, and it has since been recognised that it plays an important role in a variety of physiological and pathological processes including in cancer, wound healing and inflammation, by regulating cell proliferation, survival, migration, invasion and/or differentiation [18, 19]. Ang‐3 (mouse) and Ang‐4 (human) are considered interspecies orthologs [20]. Two of the Angs, Ang‐1 and Ang‐4, activate the Tie‐2 receptor, whereas Ang‐2 and Ang‐3 inhibit Ang‐1 induced Tie‐2 phosphorylation [21].

**FIGURE 1:**
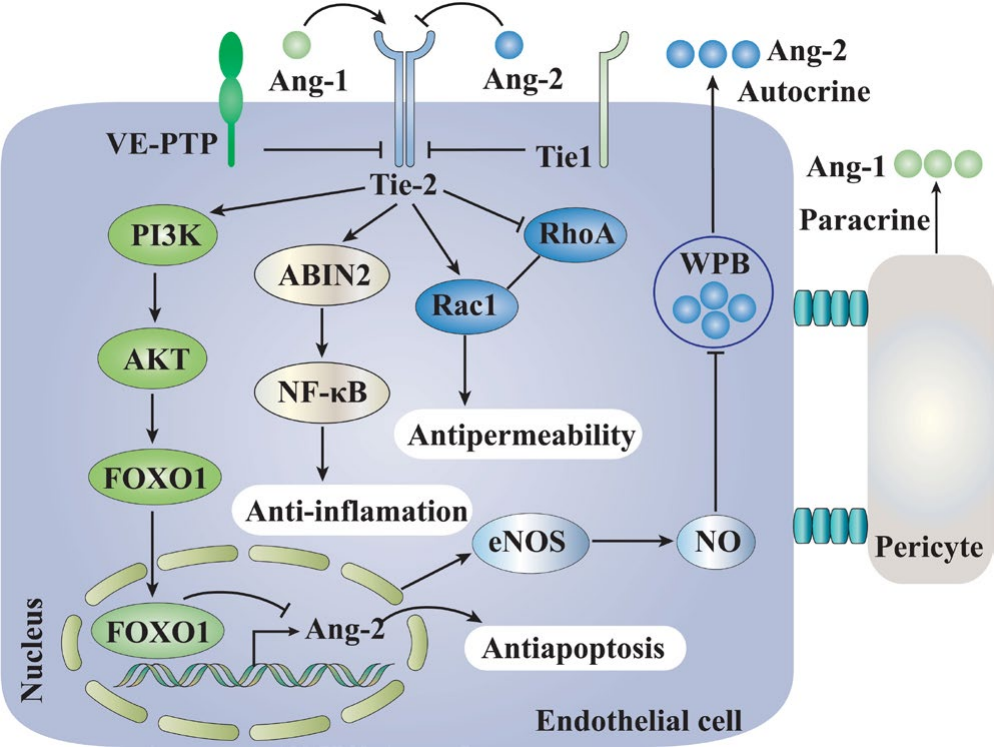
Schematic of the endothelial Tie‐2 axis signalling pathways. Angiopoietin‐1 (Ang‐1) and Angiopoietin‐2 (Ang‐2) exert the stimulation in an autocrine and/or paracrine manner. Angiopoietin‐1 (Ang‐1) ligation phosphorylates and thereby activates the Tie‐2 receptor, which promotes an anti‐inflammatory signal via inhibition of surface adhesion molecule expression and the transcription factor NF‐κB. In addition, the PI3K/Akt pathway promotes an anti‐apoptotic, pro‐survival signal. The antipermeability effects are induced by maintaining the formation of the cytoskeletal architecture. Upon stimulation, endothelial cells release prestored Ang‐2 from Weibel‐Palade bodies into the circulation. Ang‐2 competitively antagonises positive Ang‐1/Tie‐2 signalling, dramatically activating the endothelial cell and priming it for further inflammatory stimuli. Ang‐1, angiopoietin‐1; Ang‐2, angiopoietin‐2; PI3K/Akt, phosphatidylinositide 3‐kinases/activate the serine/threonine kinase.

Ang‐1 is the canonical agonist ligand for the Tie‐2 and is expressed by platelets, mesenchymal cells, vascular smooth muscle cells and pericytes. Similar to Ang‐1, Ang‐2 binds to the Tie‐2 receptor with the same binding affinity to prevent Ang‐1‐induced phosphorylation of Tie‐2, which is responsible for Ang‐1's angiogenic activity. The matrix‐bound Ang‐1 provides a constant stimulus for Tie‐2 activation in the quiescent state. Platelets store large quantities of Ang‐1 protein, releasing it from granules under the influence of inflammatory modulators [22, 23, 24]. Ang‐1 protects organs from edema and enhances survival in a growing list of infections. Ang‐2, on the other hand, potentiates leakage and inflammation such that manoeuvers to reduce Ang‐2 action are protective for the organism [25, 26]. Despite significant primary sequence homology to Ang‐1, Ang‐2 is a context‐dependent antagonist of the Tie‐2 receptor, in contrast to the stabilising actions of Ang‐1 [27, 28]. Ang‐2 is expressed exclusively by endothelial cells, stored in Weibel‐Palade bodies (WPBs) and released during hypoxia and inflammation [29, 30]. These studies have advanced our understanding of the Ang–Tie‐2 axis in the contexts of infection and sepsis. The major Ang–Tie‐2 axis signalling pathways known to be activated by Ang‐1 and Ang‐2 are summarised in Figure 1.

## 3 Tie‐2 Activation Maintains Endothelial Homeostasis

Tie‐2 is expressed in both active and quiescent endothelial cells and is necessary for mature vasculature and barrier function. Ang–Tie‐2 axis signalling is primarily involved in the maintenance of endothelial function and is essential for endothelial homeostasis. Ang‐2 acts as an antagonist to Ang‐1 at the Tie‐2 receptor on the endothelial cell surface. While Ang‐1 promotes vascular stability and preserves cell–cell contacts, Ang‐2 acts in opposition to these effects [31, 32, 33]. This cell‐specific distribution suggests their distinct roles in sepsis.

In the quiescent state, Ang‐1 binds to Tie‐2, an event that clusters individual Tie‐2 molecules into a cross‐phosphorylating complex. Downstream of phosphorylated Tie‐2, the second messenger Akt is activated, preventing endothelial apoptosis and enhancing cellular quiescence [14, 34]. Ligation of Ang‐1 to Tie‐2 leads to phosphorylation of the intracellular tyrosine cleavage domain of the receptors, which promotes endothelial cell migration and survival mainly through the canonical phosphatidyl‐inositol‐3‐kinase (PI3K)/Akt pathway [35, 36, 37]. Forkhead transcription factor (FOXO) is involved in inducing endothelial apoptosis and regulating the expression of several endothelial genes responsible for vascular destabilisation and remodelling, including Ang‐2. This inhibitory effect of Ang‐1 on FOXO is consistent with its role in the promotion of vascular quiescence [14, 38]. Besides cell survival, PI3Ks are important for several other cellular actions, such as regulating gene expression, activating endothelium migration and suppressing inflammation [39, 40, 41]. The anti‐inflammatory properties of Ang–Tie‐2 signalling are promoted by inhibiting nuclear factor kappa light chain enhancer of activated B cells (NF‐κB)‐mediated expression of leukocyte adhesion molecules such as intercellular adhesion molecule‐1 (ICAM‐1) and vascular cell adhesion molecule‐1 (VCAM‐1) [42, 43]. The antiapoptotic effects of Ang‐1 involve the phosphatidylinositol 3‐kinase (PI3K)/Akt signalling pathway. In response to Ang‐1, the PI3K regulatory subunit p85 is recruited to a phosphorylated tyrosine residue in the intracellular domain of Tie‐2. In endothelial cells, stimulation of Akt by Ang‐1 leads to phosphorylation and inhibition of the FOXO via activated PI3K [44, 45]. In addition, Ang‐1 signalling strongly activates Rac1, thereby shifting the RhoA‐Rac1 balance towards Rac1, which morphologically results in cortical F‐actin formation, promoting structural integrity of the cytoskeletal architecture. Thus, Ang‐1‐mediated Tie‐2 phosphorylation has anti‐apoptotic, anti‐inflammatory and anti‐permeability effects and maintains the quiescent state of the endothelial function.

Under pathological conditions, inhibition of Ang–1‐Tie‐2 signalling inhibits activation of the canonical PI3K/Akt pathway and induces FOXO1 activation, leading to Ang‐2 activation and compensation of Ang‐1 activity [14, 39, 46]. The pre‐formed Ang‐2 protein is stored in the WPBs so that Ang‐2 is readily available for secretion after the endothelial cells are stressed by triggers such as hypoxia, TNF‐α, turbulent flow and thrombin [47]. This Ang‐2 acts in an autocrine or paracrine manner to displace Ang‐1, thereby deactivating Tie‐2 signalling [48]. When endothelial cells are stimulated by pro‐inflammatory cytokines and VEGF, Ang‐2 expression and secretion by WPBs are upregulated, thereby establishing a Tie‐2 autocrine/paracrine regulatory mechanism [49, 50]. Taken together, the balance between Ang‐1 and Ang‐2 is tipped in favour of Ang‐2, as infection increases the expression of Ang‐2 and its release from the WPBs. Subsequently, increased Ang‐2–Tie‐2 binding blocks Tie‐2 activation and contributes to endothelial cell destabilisation. Biomarkers expressed on activated endothelial cells are summarised in Table 1.

**TABLE 1:** Biomarkers expressed on activated endothelial cells in sepsis.

Endothelial function	Biomarkers	Ligands	Effects
Vasoreactivity	Tie‐2	Angiopoietins	Regulating of endothelial cell angiogenesis and permeability
	ACE	Angiotensin	Catalysing the conversion of angiotensin I to angiotensin II for the constriction of the blood vessels and the increase in the permeability of the blood vessels
Inflammation	Endoglin	TGF‐β	Essential for angiogenesis and associated with an infiltrate of inflammatory cells
	ANGPTLs	Leukocyte immunoglobulin like receptors	Regulating angiogenesis and accelerating vascular inflammation
Leukocyte recruitment	ICAM‐1	ICAM‐1	Attracting the adhesion and movement of leukocytes to endothelial cells
	VCAM‐1	VLA‐4 integrin	Attracting the adhesion and movement of leukocytes to endothelial cells
	PECAM‐1	CD31	Enhancing endothelial cell–cell junctions for improvement of endothelial barrier properties and modulation of leukocyte adhesion and migration on endothelial cells
	E‐selectin	Sialyl‐Lewis X antigen and other carbohydrates	Regulating the movement of leukocytes on endothelial cells
	P‐selectin	Carbohydrate determinants on selectin ligands	Modulating the adhesion and movement of leukocytes on the endothelial cells
Angiogenesis	VEGFR‐1 (Flt‐1)	VEGF	Facilitating the migration, the hyperpermeability and the angiogenesis of the endothelial cells
	VEGFR‐2 (Flk‐1)	VEGF	Promoting the progression of some types of cancer
Coagulation	vWF	Factor VIII, platelet glycoprotein, heparin and collagen.	Regulating angiogenesis, proliferation, migration and the release of Ang‐2

Abbreviations: ACE, angiotensin converting enzyme; Ang‐2, angiopoietins‐2; ANGPTL, angiopoietin like proteins; Flk‐1, foetal liver kinase‐1; Flt‐1, fms‐like tyrosine kinase‐1; ICAM‐1, intercellular adhesion molecule‐1; PECAM‐1, platelet endothelial cell adhesion molecule‐1; TGF‐β, transforming growth factor‐β; Tie‐2, tyrosine kinase receptor with immunoglobulin and epidermal growth factor homology domains‐2; VCAM‐1, vascular cell adhesion molecule‐1; VEGF, vascular endothelial growth factor; VEGFR, vascular endothelial growth factor receptor; vWF, von Willebrand factor.

## 4 Tie‐2 Axis Dysregulation Contributes to Endothelial Injury

Proteomics studies have confirmed that activated Tie‐2 inhibits Ang‐2 gene transcription by sequestering the transcription factor FOXO1 in the endothelial cytoplasm, thereby enhancing the switch from quiescence to perturbation. Thus, the endothelial cell begins to synthesise more Ang‐2 through FOXO1‐mediated gene transcription when Tie‐2 phosphorylation is turned off by the rapid release of pre‐formed Ang‐2 [46, 51]. Although many reliable biomarkers have been investigated to monitor endothelial activation and injury in an attempt to find diagnostic and therapeutic tools [5, 52, 53], there are no specific therapies to treat sepsis due to its complex pathophysiology.

Patients with sepsis already suffer from hypotension, shock and impaired perfusion; increased loss of fluid into the intravascular space further impairs perfusion and increases mortality [54, 55]. The pathophysiological basis of vascular leakage in acute inflammation is still controversial, but studies have provided strong visual evidence of two related processes: (1) the contraction of endothelial cells in venules and (2) the development of gaps between adjacent cells through which water and large molecules can escape from the blood stream [52, 56, 57]. The underlying mechanism is that Ang‐2 is upregulated under pathological conditions and acts as a context‐dependent agonist/antagonist of the Ang–Tie‐2 axis, causing vascular destabilisation and sensitising blood vessels to the effects of vascular endothelial growth factor‐A (VEGF‐A) [58, 59]. Ang‐2 and VEGF‐A synergistically drive vascular leakage, neovascularisation and inflammation. Then Ang‐2 competes with Ang‐1 and the vascular endothelial protein tyrosine phosphatase (VE‐PTP) to interfere with the Ang–Tie‐2 axis, resulting in vascular leakage [60, 61]. Furthermore, inhibition of Ang‐2 or Tie‐2 activation completely abolished endothelial glycocalyx damage. Mechanistically, sepsis‐induced degradation of the endothelial glycocalyx required loss of its major component, heparan sulphate, by the heparan sulphate‐specific enzyme heparanase, which was suppressed by Tie2 activation. However, Tie‐2 activation, but not Ang‐2 inhibition, initiated after septic or enzymatic injury, induced rapid endothelial glycocalyx repair [62, 63]. These data suggest that endothelial glycocalyx breakdown in human sepsis is mediated by Tie‐2 deactivation by Ang‐2. Tie‐2 activation appears to accelerate endothelial glycocalyx recovery and may be a promising therapeutic target in human sepsis.

Ang‐2 levels were upregulated in patients with stroke, a cerebrovascular disease associated with disruption of the blood–brain barrier. Expression analysis of brain endothelial cells from Ang‐2 gain‐of‐function mice showed a downregulation of tight adherens junction molecules and increased caveolin‐1, a vesicular permeability‐related molecule [64]. In septic mice, exposure to LPS also increased Ang‐2 while inhibiting Ang‐1‐Tie‐2 expression with a reduced pericyte/endothelial coverage [65]. Reduced pericyte coverage and defective intra‐endothelial junctions with increased vesicles and reduced/disrupted glycocalyx were confirmed by immunohistochemistry and electron microscopy analyses [66]. In an in vivo study, Tie‐2 expression was found to be severely reduced in several mouse models of critical illness, leading to increased vascular permeability and mortality [67]. Preclinical evidence suggests that modulating the Ang–Tie‐2 pathway restores vascular stabilisation and reduces inflammation [28, 68]. These findings are in line with the pathological progression of sepsis, which includes hypoalbuminemia, tissue edema, acute pulmonary edema and even septic shock. These studies suggest that the Ang–Tie‐2 axis may be both a target (for treatment) and a biomarker (for indication of treatment) for sepsis management. Biomarkers of endothelial barrier dysfunction in sepsis are listed in Table 2.

**TABLE 2:** Biomarkers of endothelial injury in sepsis.

Endothelial function	Biomarkers	Effects	Clinical implications
Vasoreactivity	Endothelin‐1	Endothelin‐1 is a vasoconstrictor by reduce the production of NO.	Endothelin‐1 is significantly elevated during endothelial dysfunction.
Permeability	Occludin	Occludin is a major component of the tight junction of epithelial and/or endothelial barriers.	Occludin levels increase in various pathological conditions, such as HIV, cancer, neuroinflammation and sepsis.
	Syndecan‐1	Syndecan‐1 is released in endothelial glycocalyx damage.	Circulating syndecan‐1 levels are associated with the severity of sepsis, acute kidney injury, need for intubation and mortality.
	Endocan	Endocan is a soluble dermatan sulphate proteoglycan in endothelial cells.	Endocan is involved in important processes like cell adhesion in inflammation and cancer progression.
	Claudin‐5	Claudin‐5 can modulate the permeability of tight junctions.	Serum levels of claudin‐5 are correlated with severe plasma leakage.
	Cadherin‐5	Cadherin‐5 is a main component of adherens junction.	Vascular endothelial cadherin deficiency results in increased permeability.
	ZO‐1	ZO‐1 is a component of tight junction proteins.	ZO‐1 has been linked to inflammatory diseases and cancer.
Inflammation	Ang‐1/2	Ang‐1 and 2 are important mediators of angiogenesis.	Ang‐1/2 dysfunction can be the cause of inflammation, tumours and restenosis.
	ANGPTLs	ANGPTLs can regulate angiogenesis and mediate inflammation.	ANGPTLs accelerate vascular inflammation, leading to endothelial dysfunction and atherosclerosis development.
Leukocyte recruitment	sICAM‐1	sICAM‐1 can bind to leukocyte adhesion molecules, facilitating leukocyte migration and adhesion to target structures.	sICAM‐1 is elevated in endothelial dysfunction and is a promoter of the inflammatory response.
	sVCAM‐1	sVCAM‐1 participates in adhering and migrating blood‐borne leukocytes to the vascular intima.	sVCAM‐1 is a marker for endothelial cell activity or injury.
	sFlt‐1	sFlt‐1 plays a key role in maintaining the balance of vascular growth and is a soluble antagonist of VEGF.	sFlt‐1 is correlated with markers of inflammation, endothelial function and cardiac stress or injury.
	sE‐Selectin	E‐selectin is a recognised marker of endothelial activation.	sE‐selectin expression by endothelial cells is critical for leukocyte recruitment during inflammation.
Coagulopathy	TF	TF exposure attracts interaction with FVII and FX, activating both clotting cascade.	TF can be both an activator of the coagulation cascade and an enhancer of vascular permeability.
	PAI‐1	PAI‐1 is a key regulator of the physiological balance between thrombosis and fibrinolysis and is the major negative regulator of plasminogen activation.	High levels of PAI‐1 have been associated with a variety of cardiovascular diseases.
	Antithrombin	Antithrombin is the active anticoagulant used in heparin therapy to block thrombin, factor 10a, and to a lesser extent factors 9a and 11a.	Heparin‐type molecules produced by endothelial cells interact with AT on the vessel wall and prevent coagulation.
	Thrombomodulin	Thrombomodulin is a transmembrane glycoprotein of the endothelial cells that plays a central role in the modulation of the natural anticoagulant system.	Thrombomodulin is able to block thrombin, thereby inactivating the procoagulant signalling pathway and downstream pro‐inflammatory responses.

Abbreviations: Ang, angiopoietin; ANGPTLs, angiopoietin like proteins; PAI‐1, plasminogen activator inhibitor‐1; sE‐Selectin, soluble E‐selectin; sFlt‐1, soluble fms‐like tyrosine kinase‐1; sICAM‐1, soluble intercellular adhesion molecule‐1; sVCAM‐1, soluble vascular cell adhesion molecule‐1; TF, tissue factor; ZO‐1, zonula occludens‐1.

## 5 Tie‐2 Axis Dysfunction Accelerates the Development of DIC

Ang‐2 is upregulated under pathological conditions and acts as a context‐dependent agonist/antagonist of the Ang‐1‐Tie2 axis, causing vascular destabilisation. The loss of Tie‐2 signalling releases a key ‘brake’ on the endothelium, allowing it to rapidly transition to the activated phenotype, with weakened adherence junctions, expression of leukocyte adhesion molecules such as ICAM‐1 and VCAM‐1, and increased procoagulant proteins on the luminal surface [69, 70, 71]. Deterioration of adhesion molecules results in edema. Each of these molecular cellular events maps to major clinical features of sepsis such as acute respiratory distress syndrome (ARDS) disseminated intravascular coagulation (DIC). Reduced Tie‐2 activation during sepsis is the result of multiple perturbations of the Tie‐2 axis, including decreased expression of both Tie‐2 and Ang‐1, the generation of soluble Tie receptors, and the antagonistic activity of Ang‐2 [31, 72].

Induction of adhesion molecules promotes secondary inflammatory injury, and perturbation of normal hemostatic mechanisms leads to DIC [32, 73]. In the septic model, the dependence of endothelial cell responses to an inflammatory stimulus on Tie‐2 was observed in microvascular beds [42]. Furthermore, Ang‐2 levels increase and competitively bind to the Tie‐2 receptor during inflammatory stimuli (e.g., TNF‐, LPS, hypoxia), blocking Ang‐1‐mediated phosphorylation [74]. Because Ang‐2 is less efficient than Ang‐1 at clustering Tie‐2 monomers, the net effect of Ang‐2 induction in the context of inflammation is to suppress tonic Tie‐2 signalling [27, 75]. Ang‐2 was strongly associated with traditional markers of DIC, including platelet count, but more accurately predicted mortality in two large independent cohorts. In septic mice, reduced Tie‐2 signalling preceded signs of overt DIC. During this early phase, intravital imaging of microvascular injury revealed excessive fibrin accumulation, a pattern remarkably mimicked by Tie‐2 deficiency even in the absence of inflammation [72].

**FIGURE 2:**
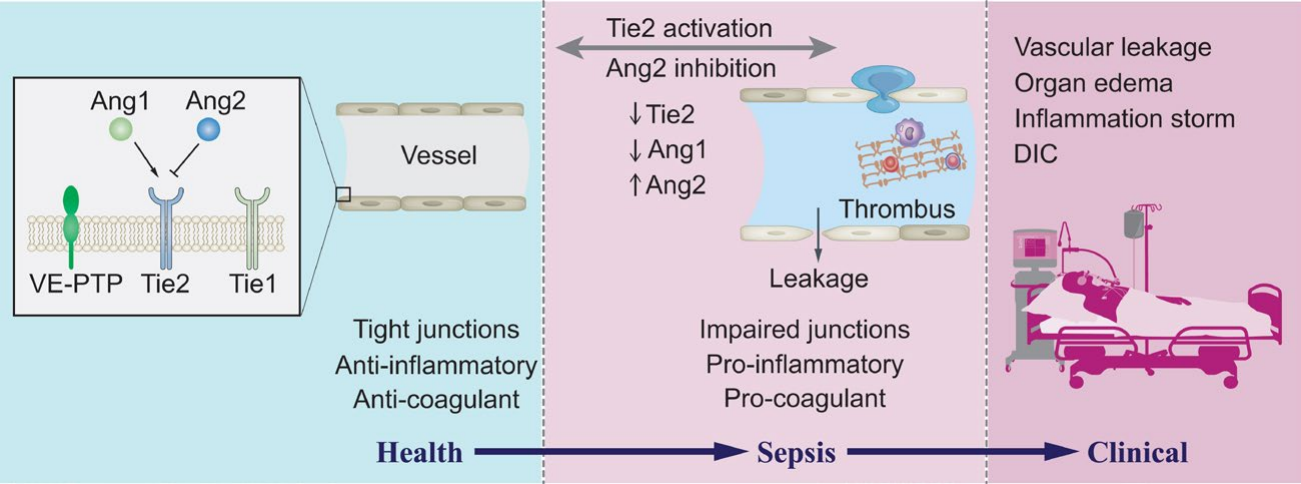
Tie‐2 axis and sepsis. Tie‐2 is activated by angiopoietin‐1 (Ang‐1) and antagonised by angiopoietin‐2 (Ang‐2). Active Tie‐2 maintains vascular quiescence, including endothelial barrier function. Both Tie‐1 and VE‐PTP antagonise Tie‐2 signalling. During sepsis, the imbalance of Ang–Tie‐2 axis effectors leads to signalling inhibition, promoting vascular leakage, inflammation and thrombosis. Microvascular damage during sepsis not only promotes vascular leakage, but also contributes to inflammation and a procoagulant state. Together, the host vascular response contributes to shock, multiorgan dysfunction, systemic inflammation and disseminated intravascular coagulation (DIC). Ang‐1, angiopoietin‐1; Ang‐2, angiopoietin‐2; VE‐PTP, vascular endothelial protein tyrosine phosphatase.

The Ang–Tie‐2 axis, which maintains endothelial homeostasis, has been suggested to play an important role in sepsis‐induced DIC by counteracting systemic inflammation and thrombosis [31, 45]. Sepsis‐induced coagulopathy is the activated intravascular coagulation caused by endothelial dysfunction in sepsis. In addition to the inflammatory status of endothelial cells, Tie‐2 signalling is also important for microvascular barrier function and thrombosis. A proteomic analysis of septic patients with DIC revealed a link between the Ang–Tie‐2 pathway and coagulation in sepsis. The results showed that alterations in Tie‐2 signalling are a triggering event in septic DIC, and that restoration of Tie‐2 activation is sufficient to attenuate thrombosis [70, 72]. Therefore, the Tie‐2 axis may be a potential therapeutic target for the treatment of sepsis‐induced DIC. The conceptual model of the Ang–Tie‐2 axis and sepsis is illustrated in Figure 2.

## 6 Targeting the Tie‐2 Axis as a Potential Treatment Strategy for Sepsis

Sepsis is a life‐threatening multiorgan dysfunction syndrome caused by a dysregulated host response to infection, with a mortality rate of over 25%, and has been designated a global health priority [76]. In recent years, despite appropriate source control and antimicrobial stewardship, there are currently no specific treatments for sepsis, and alternative treatment strategies are under investigation to prevent complications and improve outcomes. Rescue strategies for sepsis or septic shock may include various interventions, such as immunomodulatory therapies, extracorporeal support (e.g., ECMO) and therapies targeting specific molecular or cellular pathways involved in the pathophysiology of sepsis [53]. Therefore, it is necessary to identify more regulatory factors to provide a potential therapeutic strategy for the intervention of sepsis [31, 77]. Ang–Tie‐2 axis malfunction causes endothelial cell activation and contributes to sepsis pathogenesis [24, 78]. There is solid evidence that Ang‐2 levels correlate with surrogates of disease severity, including markers of tissue hypoperfusion such as serum lactate, renal injury, liver dysfunction, coagulopathy and markers of systemic inflammation and other clinical correlates of disease severity, including Acute Physiology and Chronic Health Evaluation II (APACHE II) score, Sequential Organ Failure Assessment (SOFA) score and ICU length of stay [77, 79]. Ang‐2 levels are also associated with predicted in‐hospital mortality when measured early in the course of sepsis [80, 81]. Disturbances in the Ang–Tie‐2 axis can lead to endothelial dysfunction and potentially contribute to the pathogenesis of sepsis.

Ang–Tie‐2 axis signal transduction represents multiple layers of regulation to make sure that every effector protein is activated at the right time and in the right place and will enable endothelial cells in multicellular organisms to respond to their complex environment [72, 82]. Drugs designed to restore the balance of Angs in mouse models of critical illness have shown to ameliorate organ damage, suggesting that the Ang–Tie‐2 system plays a role in developing organ failure [70, 74]. Observational studies in humans with critical illness have described an increase in circulating Ang‐2 levels associated with the disease; the circulating concentration of Ang‐1 is also consistently decreased [31, 83, 84]. Since evidence for an Ang‐2‐Tie‐2 feedback loop has been shown in cultured endothelial cells, the mechanisms underlying Ang‐2 induction in sepsis may be related to Tie‐2 inhibition [73, 85]. The relevance of this mechanism to endothelial inflammation remains to be elucidated. These results emphasise the importance of Tie‐2 as a stabiliser of endothelial homeostasis.

Current studies can be classified into three categories: (a) translation of Ang‐1 and Ang‐2 as clinically informative prognostic biomarkers in septic patients; (b) incorporation of Ang‐1 and Ang‐2 assays for clinical decision making in the treatment of sepsis and (c) development of engineered Ang‐1/Ang‐2 and Tie‐2 agonists or antagonists as novel therapeutics to prevent multiple organ dysfunction in life‐threatening infections, particularly sepsis. Potential therapeutic interventions targeting the Ang–Tie‐2 axis are summarised in Table 3.

**TABLE 3:** Tie‐2 axis targeted therapies of sepsis.

Treatment	Mechanism	Effects/Models	References
Targeting Ang‐1			
AdAng‐1	AdAng‐1 treatment inhibited NLRP3 inflammasome activation and inflammatory mediators, such as ICAM‐1, VCAM‐1, IL‐1β, IL‐18 and IL‐33, which could induce AKI and ALI.	AdAng‐1 improved survival and hemodynamic function, and reducing ALI and AKI in septic rats.	[86, 87]
rhAng‐1	RhAng‐1 treatment suppressed LPS and Ang‐2‐induced EC inflammation, reduced endothelial adhesion molecule expression, attenuated leukocyte infiltration in lungs and kidneys, and improved the function of HPMECs and HUVECs.	RhAng‐1 treatment preserved endothelial quiescence, inhibited ALI and suppressed alveolar simplification, significantly improving a variety of sepsis‐associated organ dysfunctions and survival time in the CLP mice model by preserving endothelial barrier function.	[88, 89, 90]
pAng‐1	Administration of pAng‐1 significantly reduced bronchoalveolar lavage neutrophil counts and the levels of proinflammatory cytokines, such as TNF‐α, IFN‐γ, IL‐6, MCP‐1 as well as MIP‐2.	Administration of pAng‐1 improved both alveolar inflammation and permeability in the ALI mice model.	[88, 91]
Ang‐1 variant	Ang‐1^A451D^ increased the affinity of Ang‐1 for Tie‐2, promoted Tie‐2 phosphorylation and enhanced endothelial cell migration and tube formation.	The variant Ang‐1^A451D^ reduced inflammatory cytokines and attenuated organ damage in septic mice.	[92]
Targeting Ang‐2			
Ang‐2 antibody	Ang‐2 antibody treatment prevents increased microcirculatory permeability, pericytes loss and capillary rarefaction in coronary microcirculation.	Ang‐2 antibody treatment improved the hemodynamic function and survival of septic mice.	[93, 94]
Ang‐2 siRNA	Ang‐2 siRNA decreased the transcription of pulmonary interleukin‐6, the expression of intercellular adhesion molecules, the infiltration of neutrophils and vascular leakage.	Ang‐2 siRNA improved survival and reduced the severity of illness in mice with CLP‐ or LPS‐induced sepsis, when used as either a pretreatment or a rescue intervention.	[13, 95]
Cathepsin K	Cathepsin K was necessary and sufficient to cleave Ang‐2 for the conversion of Ang‐2 from Tie‐2 agonist to antagonist.	Cathepsin K inhibition improved survival in distinct murine sepsis models, with therapeutic implications for inflammatory conditions associated with Ang‐2 induction.	[96]
Targeting Tie‐2			
Tie‐2 siRNA	Tie‐2 siRNA improved endothelial permeability, cell survival and claudin‐5 expression, promoting changes in the cytoskeleton, matrix adhesion, cell junctions and endothelial integrity of the blood–brain barrier.	Tie‐2 siRNA contributed to blood–brain barrier protection, infarct volume reduction and amelioration of neurological deficits in rats.	[97, 98]
Vasculotide	Tie‐2 agonist Vasculotide mimicked Ang‐1 effects, which could preserve microcirculatory perfusion and reduce pulmonary vascular leakage.	Vasculotide treatment improved endothelial barrier dysfunction and survival of CLP‐induced sepsis in rat model.	[74, 99, 100]

Abbreviations: ABA, Ang‐2‐blocking antibody; AdAng‐1, adenoviral‐delivered angiopoietin‐1; AKI, acute kidney injury; ALI, acute lung injury; Ang, angiopoietin; CLP, cecal ligation and puncture; EC, endothelial cell; HDMECs, human dermal microvascular blood endothelial cells; HPMEC, human pulmonary microvascular endothelial cells; HUVECs, human umbilical vein endothelial cells; ICAM‐1, intercellular adhesion molecule‐1; IFN‐γ, interferon‐gamma; IL‐6, interleukin‐6; LPS, lipopolysaccharides; MAT, matrilin‐1; MCP‐1, monocyte chemoattractant protein‐1; MIP‐2, macrophage inflammatory protein‐2; pAng‐1, plasmid containing the human angiopoietin‐1 gene; rhAng‐1, recombinant human angiopoietin‐1; siRNA, small interfering RNA; TNF‐α, tumour necrosis factor alpha; VCAM‐1, vascular cell adhesion molecule‐1 protein expression.

## 7 Concluding Remarks and Future Perspectives

The physiologically active role of endothelial cells is to supply tissues with oxygen by synthesising and releasing relaxing and contracting factors that modulate blood flow rate. Given the pivotal function of endothelial cells in angiogenesis, the targeting and blocking of endothelial cell markers with diverse antiangiogenic agents has emerged as a highly effective therapeutic strategy in numerous pathological conditions. Although our understanding of the regulatory network of the Ang–Tie‐2 is still incomplete, the characterisation of Ang‐1/Ang‐2 and Tie‐2 has been extensively illustrated and is expected to lead to the development of new therapeutic agents and improved treatments for various clinical diseases.

Endothelial cells are among the earliest cell types to detect microbial pathogens or foreign debris in the bloodstream, and their sensing ability enables them to actively participate in both innate and adaptive immune responses. Endothelial cell activation serves as an integral part of the immune defence system; however, persistent endothelial cell activation results in inflammation and endothelial dysfunction. It is now recognised that endothelial cells facilitate effective immune responses by activating and regulating immune cells, including cytokine secretion, phagocytic function, antigen presentation, pathogen‐associated molecular patterns (PAMPs) and danger‐associated molecular patterns (DAMPs) sensing, proinflammatory, immune‐enhancing, anti‐inflammatory, immunosuppression, migration, heterogeneity and plasticity. It is thus reasonable to propose that endotheliopathy may be a key factor in the pathogenesis of sepsis, particularly in light of the evidence indicating dysregulated host responses, including exaggerated inflammation, coagulation, vascular leakage and tissue hypoperfusion. Accordingly, the utilisation of particular markers for the identification of endothelial injury has been acknowledged as a promising approach for the management of sepsis with intricate underlying mechanisms.

Tie‐2 is expressed in both active and quiescent endothelial cells and is necessary for adult vasculature and barrier function. Because of its critical function, the Tie‐2 signalling pathway is regulated at multiple levels. Angiogenesis is dynamically regulated and maintained by a dynamic balance of proliferative and cytostatic signals for endothelial cells. As demonstrated above, the characterisation of Ang‐1 and Ang‐2 and Tie‐2 has been extensively illustrated and is expected to result in the development of new therapeutic agents and improved treatments for various clinical diseases. Nevertheless, our understanding of the regulatory network on the Tie‐2 axis is far from complete. Because the receptor Tie‐2 is a dynamic player in critical illness, such as sepsis, therapeutic intervention should not solely focus on the ligands Ang‐1 and Ang‐2 but should also focus on the Tie‐2 receptor. Furthermore, as effector proteins that regulate antagonistic or similar processes will also crosstalk with each other during clinical processes, these regulatory effectors are likely to be context‐dependent and will vary in response to different signals. It is also becoming apparent that effector proteins can provide positive or negative feedback to the appropriate regulation during sepsis.

In summary, the efficacy of treatments targeting the Ang–Tie‐2 axis is likely to be context‐dependent, varying in response to different signals. This is because effector proteins that regulate antagonistic or similar processes will also engage in crosstalk during clinical processes. It can be reasonably deduced that a comprehensive understanding of the regulatory mechanisms associated with the Ang–Tie‐2 axis will facilitate the identification of potential therapeutic targets for the treatment of sepsis.

## Author Contributions

J.F. and J.L. wrote the main text of the manuscript, X. W and J.W. designed Figures 1 and 2 and prepared Table 1, 2, 3–1, 2, 3. All authors read and approved the final manuscript.

## Ethics Statement

The authors have nothing to report.

## Consent

The authors have nothing to report.

## Conflicts of Interest

The authors declare no conflicts of interest.

## Data Availability

The authors have nothing to report.
